# Next-generation sequencing dataset of bacterial communities of *Microcerotermes crassus* workers associated with Ironwood trees (*Casuarina equisetifolia*) in Guam

**DOI:** 10.1016/j.dib.2023.109286

**Published:** 2023-05-31

**Authors:** Garima Setia, Robert Schlub, Claudia Husseneder

**Affiliations:** a Department of Entomology, Louisiana State University Agricultural Center, Baton Rouge, LA, United States; b University of Guam, Cooperative Extension Service, Mangilao, Guam

**Keywords:** Social insect, Termite, Bacteria, Diversity, Taxonomic index, Metataxanomics, Amplicon sequencing, 16S

## Abstract

Ironwood trees (*Casuarina equisetifolia*) in Guam have been suffering from Ironwood Tree Decline (IWTD) since 2002. Putative plant pathogenic bacteria such as *Ralstonia solanacearum* and *Klebsiella* species were identified in the ooze of declining trees and considered to be linked to IWTD. In addition, termites were found to be significantly associated with IWTD. *Microcerotermes crassus* Snyder (Blattodea: Termitidae) was identified as a termite species that attacks ironwood trees in Guam. Since termites harbor a diverse community of symbiotic and environmental bacteria, we sequenced the microbiome of *M. crassus* workers attacking ironwood trees in Guam to assess the presence of IWTD-associated pathogens in termite bodies. This dataset contains 652,571 raw sequencing reads present in *M. crassus* worker samples collected from six ironwood trees in Guam obtained via sequencing the V4 region of the16S rRNA gene on the Illumina NovaSeq (2 × 250bp) platform. Sequences were taxonomically assigned in QIIME2 using SILVA 132 and NCBI GenBank as reference databases. Spirochaetes and Fibrobacteres were the most dominant phyla in *M. crassus* workers. No putative plant pathogens of the genera *Ralstonia* or *Klebsiella* were found in the *M. crassus* samples. The dataset has been made publicly available through NCBI GenBank under BioProject ID PRJNA883256. This dataset can be used to compare the bacterial taxa present in *M. crassus* workers in Guam to bacteria communities of related termite species from other geographical locations. In addition, this dataset can also be used to investigate the relationship between termite microbiomes and the microbiomes of ironwood trees they attack and of the surrounding soil.

**Specifications Table**

**Table:** 

Subject	Entomology and insect science
Specific subject area	Metataxanomics
Type of data	16S rRNA gene amplicon sequence reads
How the data were acquired	Bacterial 16S rRNA gene sequences were obtained via sequencing on the Illumina NovaSeq (2 × 250bp) platform and analyzed using QIIME 2 (version 2021.8) and iNEXT (iNterpolation and EXTrapolation) (version 3.0.0)
Data format	Raw, analyzed, tables, and figures
Description of data collection	Samples of *M. crassus* workers were collected from six ironwood trees (*Casuarina equisetifolia*) in Guam and shipped in 95% ethanol to LSU AgCenter. Total DNA was extracted from the whole bodies of five workers per sample (DNeasy Blood & Tissue kit (Qiagen, Germantown, MA)). The V4 region of the 16S rRNA gene was sequenced on the Illumina NovaSeq (2 × 250bp) platform following the Illumina Nextera Dilute library protocol to describe the bacterial communities using QIIME 2 version 2021.8 and iNEXT for diversity analyses and SILVA 132 and NCBI GenBank as reference databases.
Data source location	Bacterial communities described for *M. crassus* worker samples were collected from ironwood trees at six different locations on the island of Guam (Table 3).
Data accessibility	NCBI GenBank with BioProject ID PRJNA883256 [Accession Numbers: SRX17796823, SRX17796825, SRX17796826, SRX17796827, SRX17796828, SRX17796829] [https://www.ncbi.nlm.nih.gov/sra/?term=PRJNA883256+Microcerotermes].

## Value of the Data

• This dataset contributes to the investigation of Ironwood Tree Decline which is significantly associated with the presence of putative bacterial plant pathogens and termites on ironwood trees in Guam [1,2].
• *Microcerotermes crassus* workers harbor diverse bacteria and have been shown to attack ironwood trees in Guam [3]. Therefore, this dataset of bacterial communities of *M. crassus* workers associated with ironwood trees in Guam, would be useful to conservation scientists, foresters, and pest management professionals for assessing whether worker termites of this species could be vectors for IWTD pathogenic bacteria.
• This dataset describes bacterial communities present in *M. crassus* of Guam. Microbiologists and entomologists can use this dataset to compare the bacteria in *M. crassus* with those found in other similar termite species in Guam or in other geographic regions.
• This data can be utilized by microbiologists and plant pathologists to understand if there is an association between the microbiota of termites with the microbiota of the ironwood trees they are attacking or the microbiota of the soil surrounding the ironwood trees.

## 1 Objective

*Casuarina equisetifolia* (ironwood) trees in Guam are dying in large numbers due to ironwood tree decline (IWTD) since 2002 [[4], [5], [6], [7]]. Bacteria such as the bacterial wilt pathogen *Ralstonia solanacearum* and wetwood bacteria of the genus *Klebsiella* (*Klebsiella oxytoca* and *Klebsiella variicola*) were identified in the ooze of declining trees and considered as predictors of IWTD [6,7,2,8]. The presence of termites was found to be significantly associated (p<0.01) with IWTD [1] and termites are known to harbor a large bacteria diversity [9]. This dataset was generated to describe the bacterial composition of *M. crassus* termite workers collected from ironwood trees and assess if workers carry putative bacterial pathogens associated with IWTD.

## 2 Data Description

To identify the bacterial taxa present in *M. crassus* workers, the V4 variable region of the 16S rRNA was amplified using the Illumina NovaSeq platform. The links and accession numbers to the fastq files in this dataset are provided in Table 1. The bacterial sequences present in all the samples were assigned to their respective taxa using the Quantitative Insights into Microbial Ecology (QIIME2 version 2021.8) pipeline [10].

**Table 1:** Accession numbers and links for raw fastq sequences of *M.* crassus samples collected from six ironwood trees in Guam.

Sample name	SRA number	Accession link
21-187	SRR21803026	https://www.ncbi.nlm.nih.gov/sra/SRX17796829[accn]
21-179	SRR21803027	https://www.ncbi.nlm.nih.gov/sra/SRX17796828[accn]
21-178	SRR21803028	https://www.ncbi.nlm.nih.gov/sra/SRX17796827[accn]
21-177	SRR21803029	https://www.ncbi.nlm.nih.gov/sra/SRX17796826[accn]
21-163	SRR21803030	https://www.ncbi.nlm.nih.gov/sra/SRX17796825[accn]
21-152	SRR21803032	https://www.ncbi.nlm.nih.gov/sra/SRX17796823[accn]

A total of 652,571 raw sequencing reads were obtained across six *M. crassus* worker samples collected from six ironwood trees in Guam. A total of 378,976 sequence reads represented by 2,165 Amplicon Sequence Variants (ASVs) remained after quality filtering using DADA2. The removal of ASVs with no taxonomical assignment at 97% identity to references in the SILVA 132 database resulted in 231,967 sequence reads and 831 ASVs.

**Fig. 1:**
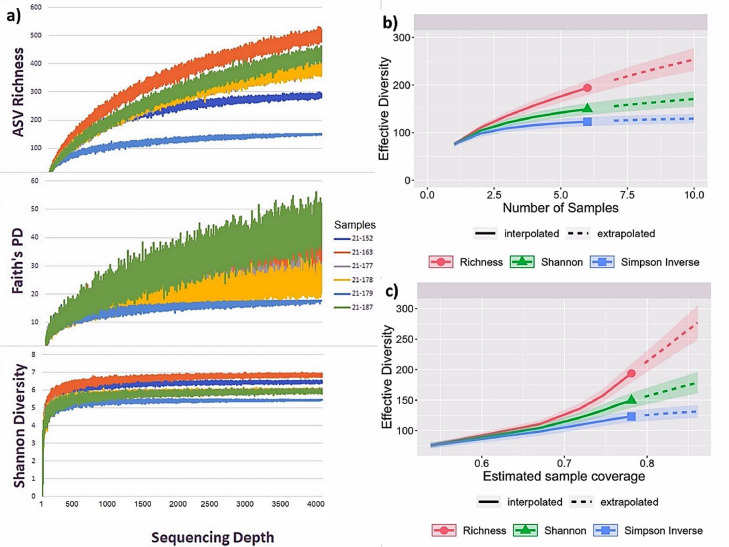
a) Sequence-based rarefaction curves of bacteria diversity showing the ASV richness, Faith's phylogenetic distance and Shannon diversity indices in six *M. crassus* worker samples plotted against sequencing depth. b) Sample-based rarefaction curves with effective bacterial diversity for different metrics plotted against the number of samples. c) Coverage-based rarefaction curves with effective diversity plotted against estimated sample coverage. Solid lines indicate intrapolation up to the actual sample size; dashed lines represent extrapolation to twice the sample size. Rarefaction was performed over the total bacteria diversity (with and without taxonomical assignment). Sequencing depth was measured as number of reads.

Rarefaction curves (Fig. 1) were plotted to assess whether sequencing depth, sample numbers, and coverage were sufficient to capture most of the bacterial diversity present in the *M. crassus* worker samples. The sequence-depth based rarefaction curves were generated by plotting sequencing depth against different alpha diversity metrics. The sequence-depth based rarefaction curves for ASV richness and Faith's phylogenetic distance (PD) between the ASVs (Fig. 1a) started to level out at a sequencing depth of around 1,500. The sequence-depth based rarefaction curves for Shannon diversity levelled out at sequencing depth of 500. The levelling out of the rarefaction curves indicated that sequencing depth of the samples captured most of the bacteria diversity within each sample.

Sample-, and coverage-based rarefaction curves (Fig. 1b, c) were generated by plotting effective diversity against number of samples and estimated sample coverage, respectively. The effective diversity measures both relative abundance and richness and is quantified by Hill numbers (parameterized by *q*). ASV richness, Shannon diversity, and Simpson diversity were quantified at q=0, 1 and 2, respectively. The sample- and coverage-based rarefaction curves were extrapolated to twice the sample size to compute the effective diversity.

The sample-based rarefaction curves (Fig. 1b) started to level out at an effective diversity of around 150 and 120 for Shannon diversity and Simpson inverse, respectively, and extrapolation did not considerably increase these values. The sample-based rarefaction curve for ASV richness continued to increase after extrapolation. However, the increase in ASV richness would be due to rare ASVs since the increase in ASV richness was not accompanied by an increase in Shannon diversity and Simpson inverse (Fig. 1b). Six samples provided around 80% sample coverage (Fig. 1c). Extrapolation of the curves to twice the sample size increased the coverage to 95%.

**Fig. 2:**
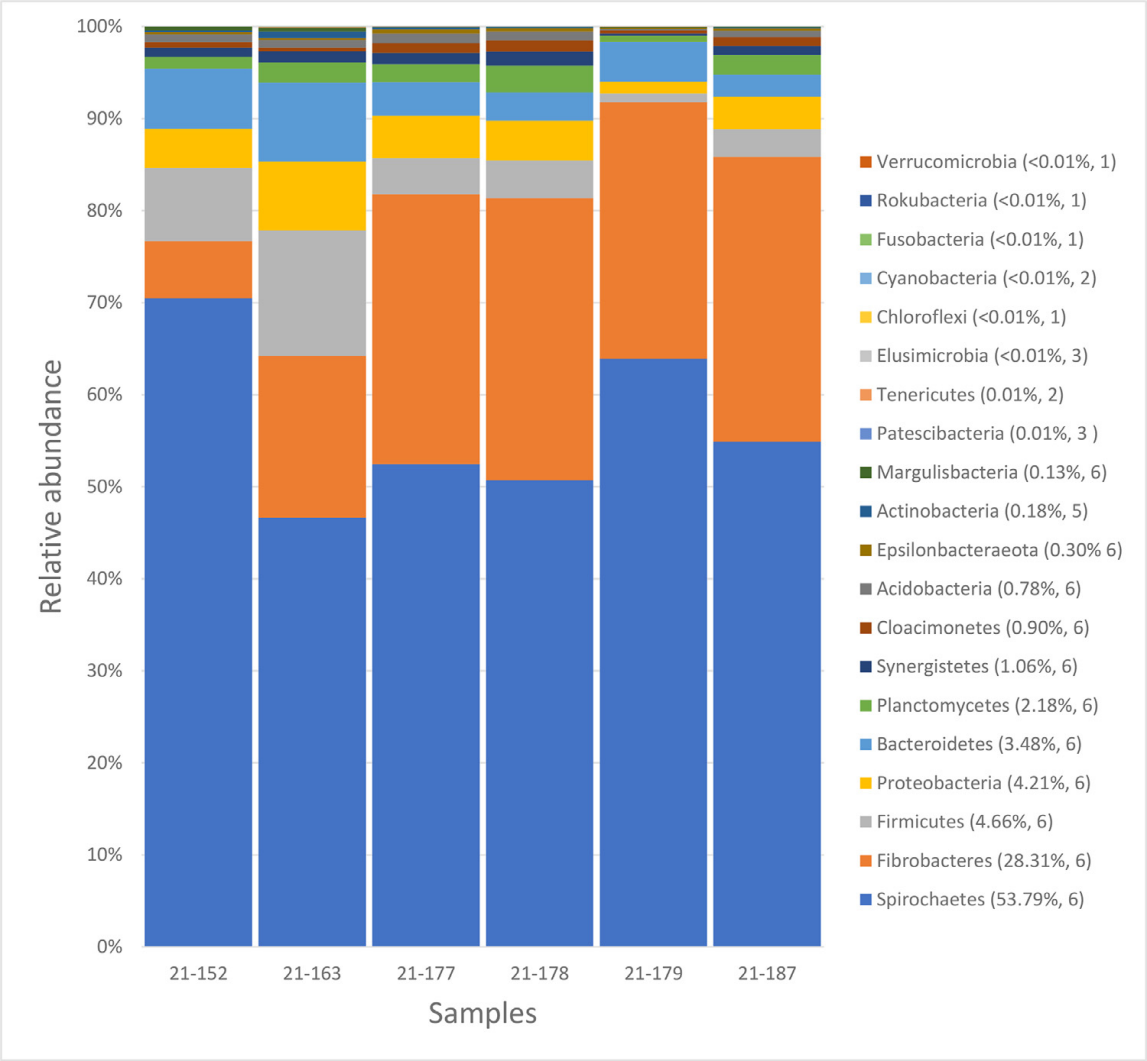
Taxa bar plots showing the relative abundance of bacterial phyla associated with six samples of *M. crassus* workers collected from ironwood trees in Guam. Legend depicts name of the phylum with its relative abundance (in %) and number of samples in which that phylum was found.

The taxonomic assignment of ASVs revealed twenty different phyla in *M. crassus* workers collected from ironwood trees in Guam including Spirochaetes (53.79%), Fibrobacteres (28.31%), Firmicutes (4.66%), Proteobacteria (4.21%), Bacteroidetes (3.48%), Planctomycetes (2.18%), Synergistetes (1.06%) and others (<2.4%) (Fig. 2). Twenty ASVs with highest number of reads were assigned to phyla Spirochaetes, Fibrobacteres, Proteobacteria, Planctomycetes, Firmicutes, Cloacimonetes, Bacteroidetes, Synergistetes, and Acidobacteria (Table 2). The ASV with the highest number of reads was assigned to an uncultured *Treponema* sp. from the phylum Spirochaetes (Table 2). None of the ASV was assigned to putative pathogens associated with IWTD in Guam such as *Ralstonia* spp. and *Klebsiella* spp.

**Table 2:** The 20 ASVs with highest number of reads across the M. crassus samples taxonomically classified according to the SILVA 132 and GenBank database.

Phylum	Order	Lowest SILVA assignment	Scientific name	Percent identity to top match in GenBank	Accession number in GenBank	Number of reads	Number of samples	Average reads per sample	Standard deviation
Spirochaetes	Spirochaetales	uncultured Treponema sp.	uncultured Treponema sp.	99.13%	AB191906.1	98,787	6	16,465	23,843
Fibrobacteres	Fibrobacterales	uncultured Chitinivibrionia bacterium	uncultured Chitinivibrionia bacterium	98.27%	AB255975.1	63,462	6	10,577	16,764
Spirochaetes	Spirochaetales	uncultured Treponema sp.	uncultured Treponema sp.	98.70%	AB191972.1	24,063	6	4,011	5,111
Proteobacteria	Rs-K70 termite group	uncultured delta proteobacterium	uncultured delta proteobacterium	100.00%	AB255924.1	7,250	6	1,208	1,538
Planctomycetes	Pirellulales	uncultured planctomycete	uncultured planctomycete	99.13%	KM651184.1	4,707	6	785	1,129
Firmicutes	Clostridiales	uncultured Eubacteriaceae bacterium	uncultured bacterium	99.13%	AB277904.1	4,334	6	722	907
Firmicutes	Clostridiales	uncultured Clostridiaceae bacterium	uncultured Clostridiaceae bacterium	100.00%	AB192029.1	2,324	5	387	840
Cloacimonetes	Cloacimonadales	uncultured bacterium	uncultured bacterium	97.40%	AB191981.1	2,097	6	350	525
Bacteroidetes	SJA-28	uncultured Chlorobi bacterium	uncultured Chlorobi bacterium	98.70%	AB192128.1	1,903	6	317	330
Fibrobacteres	Fibrobacterales	uncultured Fibrobacteres bacterium	uncultured Fibrobacteres bacterium	99.57%	AB192087.1	1,703	6	284	336
Bacteroidetes	Bacteroidales	uncultured Bacteroidales bacterium	uncultured Bacteroidales bacterium	98.27%	AB191992.1	1,528	5	255	261
Bacteroidetes	Bacteroidales	uncultured Bacteroidales bacterium	uncultured Bacteroidales bacterium	98.27%	AB191995.1	1,430	6	238	309
Firmicutes	Clostridiales	uncultured Clostridiaceae bacterium	uncultured Clostridiaceae bacterium	99.13%	AB192023.1	1,418	6	236	263
Synergistetes	Synergistales	uncultured bacterium	uncultured bacterium	98.70%	KM023943.1	971	5	162	186
Synergistetes	Synergistales	uncultured bacterium	uncultured bacterium	99.13%	AB243291.1	969	6	162	202
Acidobacteria	Holophagales	uncultured Acidobacteria bacterium	uncultured Acidobacteria bacterium	98.27%	AB192122.1	957	5	160	183
Acidobacteria	uncultured Acidobacteria bacterium	uncultured Acidobacteria bacterium	uncultured Acidobacteria bacterium	97.40%	AB192123.1	848	6	141	184
Bacteroidetes	Bacteroidales	uncultured Bacteroidetes bacterium	uncultured Bacteroidetes bacterium	99.57%	AB192003.1	763	5	127	157
Proteobacteria	Rhodospirillales	uncultured alpha proteobacterium	uncultured Alphaproteobacteria bacterium	100.00%	AB192062.1	743	5	124	163
Bacteroidetes	Bacteroidales	uncultured Bacteroidales bacterium	uncultured Bacteroidetes bacterium	98.27%	KM650310.1	723	5	121	147

## 3 Experimental Design, Materials and Methods

### 3.1 Termite collection and DNA sequencing

Workers and soldiers of *M. crassus* were collected by the team of the University of Guam in 2021 from six different ironwood trees in Guam (Table 3). Trees were separated by at least 30 meters to ensure that all the termite samples collected were from different colonies. Termite samples were preserved in 95% ethanol and sent to Louisiana State University for analysis. The soldier caste was used for morphological identification of the termite species [3]. Five workers per sample were pooled and DNA was extracted using DNeasy Blood & Tissue kit (Qiagen, Germantown, MA). We used sterile techniques throughout the DNA extraction process to minimize the risk of contamination. The concentration of extracted DNA was measured with the Invitrogen Qubit 4 Fluorometer (Thermo Fisher Scientific, Wilmington, DE) using the Qubit dsDNA BR Assay Kit (Invitrogen^TM^, Life Technologies^TM^). The DNA was sent to the University of New Hampshire Hubbard Center for Genome Studies for sequencing. The V4 region of 16S rRNA gene of the bacterial DNA was amplified using the primers 515F and 926R [11] and sequenced on the Illumina NovaSeq (2 × 250bp) platform using Illumina Nextera Dilute library protocol with a spike-in of 1% Phi X (Illumina, San Diego, CA).

**Table 3:** Location of ironwood trees from where 6 samples of *Microcerotermes crassus* termites were collected.

Sample ID	Region	*GPS coordinates*
21-187	Fort Soledad	13.2940, 144.6621
21-179	Mangilao Golf Course	13.4683, 144.8482
21-178	Mangilao Golf Course	13.4683, 144.8482
21-177	Mangilao Golf Course	13.47025, 144.84740
21-163	Padre Palmo Park, East Agana	13.477254, 144.75693
21-152	UOG Inarajan Station	13.2830, 144.7560

### 3.2 Bioinformatics and statistical analysis

Bioinformatic analysis was performed using QIIME2 version 2021.8 pipeline [10]. Demultiplexed fastq sequences were obtained after sequencing from the University of New Hampshire Hubbard Center for Genome Studies. Primers and chimera sequences were removed and Phred quality scores of the sequences were checked using DADA2 [12]. All the sequences were of good quality (Phred quality score > 30); therefore, no trimming was required. Sequence reads of 251 nucleotide length were obtained and forward reads were used for further analysis. Sequence depth based-rarefaction curves were plotted after subsampling the sequence reads to the number of sequences in the sample with the lowest sequencing depth of 4,118 using the QIIME 2 pipeline. Sample size- and coverage-based rarefaction curves were plotted using R package iNEXT (iNterpolation/ EXTrapolation) [13]. The sequence reads or ASVs obtained after DADA2 quality filtering were assigned to their respective taxa by comparing them to the SILVA 132 reference database [14] using the consensus method in BLAST at a 97% pairwise identity cutoff. The ASVs that were not assigned to any taxonomic group were removed from the dataset before generating taxa bar plots showing relative abundance of ASVs at the phylum level. The taxonomical assignments of the top 20 ASVs with the highest number of reads were cross-checked against references in NCBI GenBank database (2021) by performing BLAST [15]. Codes for analysis in this manuscript are available at https://github.com/garima-setia/Microcerotermes-crassus.

## Ethics Statements

Not applicable.

## CRediT Author Statement

**Husseneder, Claudia**: Conceptualization, Supervision, Writing – review & editing; **Schlub, Robert**: Sample Collection, Writing – review & editing; **Setia, Garima:** Writing – original draft, Data Analysis.

## Declaration of Competing Interest

The authors declare that they have no known competing financial interests or personal relationships that could have appeared to influence the work reported in this paper.

## Data Availability

Next-generation sequencing dataset of bacterial communities of Microcerotermes crassus workers associated with Ironwood trees (Casuarina equisetifolia) in Guam (Original data) (NCBI).
